# Phosphoproteomic analysis of thrombin- and p38 MAPK-regulated signaling networks in endothelial cells

**DOI:** 10.1016/j.jbc.2022.101801

**Published:** 2022-03-04

**Authors:** Olivia Molinar-Inglis, Jacob M. Wozniak, Neil J. Grimsey, Lennis B. Orduña-Castillo, Norton Cheng, Ying Lin, Monica L. Gonzalez Ramirez, Cierra A. Birch, John D. Lapek, David J. Gonzalez, JoAnn Trejo

**Affiliations:** 1 Department of Pharmacology, School of Medicine, University of California, San Diego, La Jolla, California, USA; 2 Biomedical Sciences Graduate Program, University of California, San Diego, La Jolla, California, USA; 3 Department of Pharmaceutical and Biomedical Sciences, University of Georgia, College of Pharmacy, Athens, Georgia, USA; 4 Skaggs School of Pharmacy and Pharmaceutical Sciences, University of California, San Diego, La Jolla, California, USA

**Keywords:** α-catenin, ERK1/2, GPCR, inflammation, protein kinase, AGC, automatic gain control, CDK, cyclin-dependent kinase, DMSO, dimethylsulfoxide, ERK, extracellular signal–regulated protein kinase, GPCR, G protein–coupled receptor, GPS, group-based prediction system, MAPK, mitogen-activated protein kinase, MLC, myosin light chain, MS, mass spectrometry, NF-kB1, nuclear factor kappa B subunit 1, PAR1, protease-activated receptor-1, PKD, protein kinase D, TAB1, transforming growth factor-β-activated kinase 1-binding protein 1, TAB2, transforming growth factor-β-activated kinase 1-binding protein 2, TMT, tandem mass tag

## Abstract

Endothelial dysfunction is a hallmark of inflammation and is mediated by inflammatory factors that signal through G protein–coupled receptors including protease-activated receptor-1 (PAR1). PAR1, a receptor for thrombin, signals *via* the small GTPase RhoA and myosin light chain intermediates to facilitate endothelial barrier permeability. PAR1 also induces endothelial barrier disruption through a p38 mitogen-activated protein kinase–dependent pathway, which does not integrate into the RhoA/MLC pathway; however, the PAR1-p38 signaling pathways that promote endothelial dysfunction remain poorly defined. To identify effectors of this pathway, we performed a global phosphoproteome analysis of thrombin signaling regulated by p38 in human cultured endothelial cells using multiplexed quantitative mass spectrometry. We identified 5491 unique phosphopeptides and 2317 phosphoproteins, four distinct dynamic phosphoproteome profiles of thrombin-p38 signaling, and an enrichment of biological functions associated with endothelial dysfunction, including modulators of endothelial barrier disruption and a subset of kinases predicted to regulate p38-dependent thrombin signaling. Using available antibodies to detect identified phosphosites of key p38-regulated proteins, we discovered that inhibition of p38 activity and siRNA-targeted depletion of the p38α isoform increased basal phosphorylation of extracellular signal–regulated protein kinase 1/2, resulting in amplified thrombin-stimulated extracellular signal–regulated protein kinase 1/2 phosphorylation that was dependent on PAR1. We also discovered a role for p38 in the phosphorylation of α-catenin, a component of adherens junctions, suggesting that this phosphorylation may function as an important regulatory process. Taken together, these studies define a rich array of thrombin- and p38-regulated candidate proteins that may serve important roles in endothelial dysfunction.

Vascular endothelial dysfunction is caused by various inflammatory mediators that signal through G protein–coupled receptors (GPCRs) (1, 2), such as protease-activated receptor-1 (PAR1) (3). PAR1, a receptor for thrombin, signals through a p38 mitogen-activated protein kinase (MAPK)–dependent pathway to promote endothelial barrier permeability *in vitro* and vascular leakage *in vivo* (4, 5). However, the thrombin/PAR1-stimulated p38 signaling pathway does not integrate into the RhoA/myosin light chain (MLC) pathway (6). Our recent studies demonstrated that inhibition of p38 failed to block RhoA activation and phosphorylation of MLC induced by thrombin, and conversely inhibition of RhoA signaling failed to block thrombin-stimulated p38 activation (6). We further showed that p38 signals in part *via* downstream effector kinases, MAPKAPK2 (MK2) and MAPKAPK3 (MK3) to phosphorylate heat shock protein 27 (HSP27), which acts in a counter-regulatory role to promote endothelial barrier recovery after proinflammatory GPCR-induced disruption (6). We hypothesize that GPCR-activated p38 MAPK impinges on multiple intersecting signaling pathways that regulate endothelial dysfunction. Yet, the p38-regulated intracellular signaling pathways induced by GPCRs that control endothelial barrier disruption and recovery as well as other inflammatory responses are not well defined.

The p38 kinase family includes four members, the ubiquitously expressed p38α and p38β isoforms, and the p38γ and p38δ isoforms that exhibit tissue-specific expression. All four p38 family members are activated by dual phosphorylation through a canonical three-tiered kinase cascade mediated by a MAP2K, which is phosphorylated by an upstream MAP3K. In addition to the three-tiered kinase cascade, p38α can be activated by autophosphorylation induced by transforming growth factor-β-activated kinase 1-binding protein 1 (TAB1). Activation of p38α by TAB1 binding has been shown to function *in vivo* in multiple settings including myocardial ischemia, skin inflammation, and T cell senescence (7, 8, 9, 10). We previously showed that several endothelial GPCR agonists including thrombin, histamine, ADP, and prostaglandin E2 stimulate p38α activation *via* a TAB1- and TAB2-dependent pathway in endothelial cells to promote vascular inflammation (4, 5). However, the downstream effectors of GPCR-stimulated p38 signaling are largely unknown.

In contrast to canonical p38 activation, noncanonical activation of p38 by endothelial GPCRs occurs primarily from endosomes. We showed that ubiquitination of PAR1 by activated NEDD4-2 E3 ubiquitin ligase initiates recruitment of TAB2, a structural homolog of TAB1, that binds to TAB1 and triggers p38 activation (4, 11). While activation of NEDD4-2 is initiated by G protein signaling at the plasma membrane, the ubiquitinated PAR1–TAB2–TAB1 complex accumulates on endosomes and propagates prolonged p38 signaling in the cytoplasm. The distinct spatial and temporal regulation of p38 activity by endothelial GPCRs is important for defining the cellular responses to p38 signaling, however, the p38 targets of phosphorylation that enable specific thrombin-induced cellular responses are not known.

To elucidate the pathways and proteins that engender thrombin-activated PAR1-stimulated p38 signaling in human endothelial cells, we used a quantitative phosphoproteomic approach. Here, we report the global thrombin phosphoproteome regulated by p38 using tandem mass tag (TMT) mass spectrometry (MS). Our study unveils four unique dynamic phosphoproteome profiles of thrombin signaling regulated by p38 and the identification of multiple enriched biological functions associated with microtubules, focal adhesions, stress fiber, endocytosis, Rho, small GTPases, and cell–cell adherens junctions, which function as key modulators of endothelial dysfunction. We also identified distinct sets of kinases and subsets of candidate proteins predicated to mediate thrombin-stimulated p38-induced cellular responses. In validation studies, we show that p38 negatively regulates basal phosphorylation of extracellular signal–regulated protein kinase 1/2 (ERK1/2) in endothelial cells resulting in amplified ERK1/2 phosphorylation after thrombin stimulation. We further show that p38 uniquely mediates phosphorylation of α-catenin, a key component of adherens junctions, which has not been previously reported. Together, these studies identify an important array of proteins and pathways that specify thrombin-stimulated p38 signaling in endothelial cells.

## Results

### Quantitative phosphoproteomic workflow of thrombin-induced p38 MAPK dependent signaling

**Figure 1:**
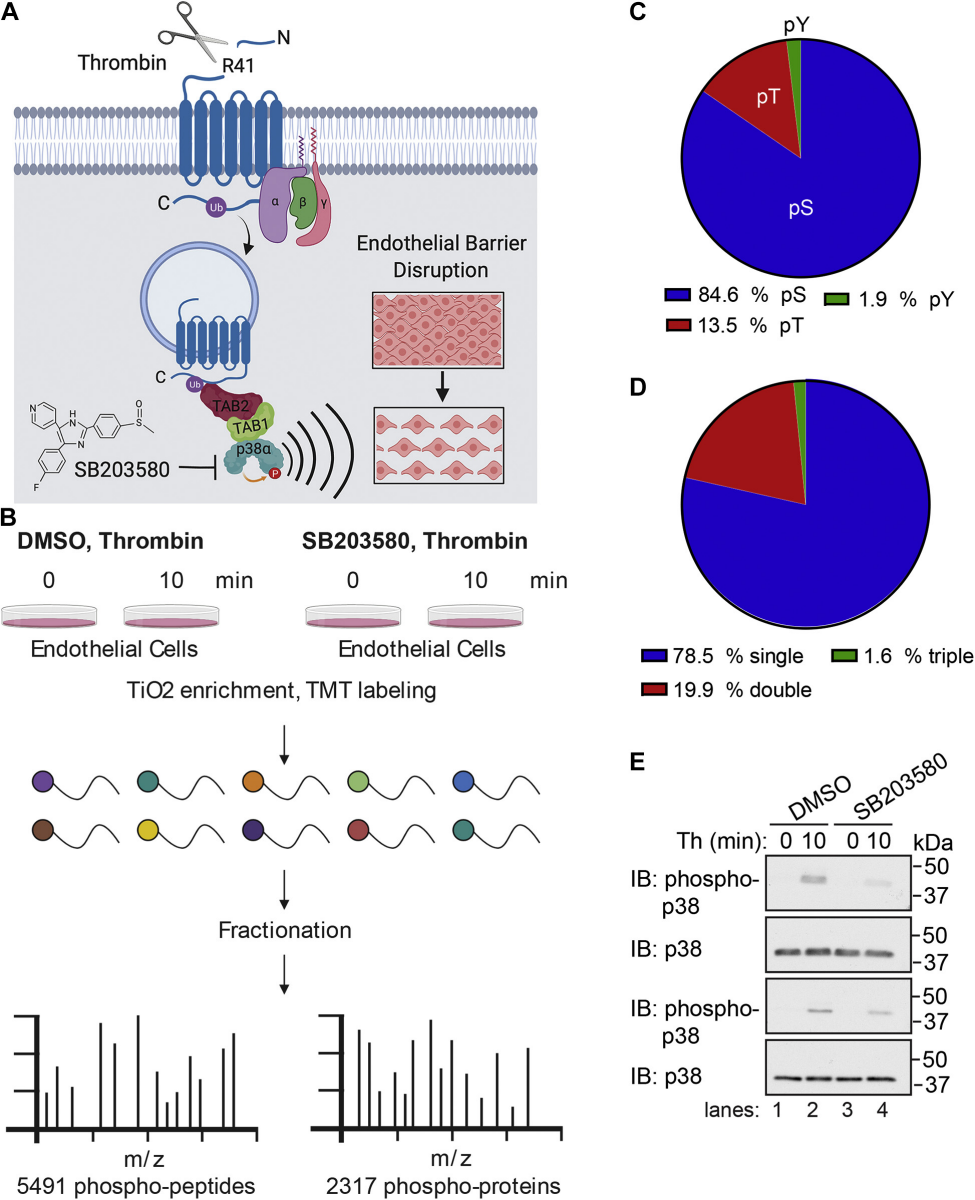
**Quantitative phosphoproteomic analysis of thrombin-induced p38 MAPK signaling in endothelial cells.***A*, thrombin activates PAR1 through proteolytic cleavage at the N-terminus R41 site and initiates heterotrimeric G protein coupling that drives ubiquitin-mediated recruitment of TAB1 and TAB2 to promote p38 activation from endosomes and disruption of endothelial barrier. SB203580 inhibits thrombin-stimulated p38 activation and endothelial barrier disruption. *B*, endothelial cells pretreated with SB203580 or DMSO were stimulated with thrombin and processed for quantitative mass spectrometry. *C*, pie chart of the phosphosite distribution. *D*, pie chart of the number of phosphosites per peptide distribution. *E*, cell lysates from endothelial cells pretreated with SB203580 or DMSO and stimulated with thrombin (Th) were immunoblotted for p38 and p38 phosphorylation performed in duplicate. MAPK, mitogen-activated protein kinase; PAR1, protease-activated receptor-1; TAB1, transforming growth factor-β-activated kinase 1-binding protein 1.

Thrombin stimulates endothelial proinflammatory responses including endothelial barrier disruption through cleavage of an N-terminal arginine (R)-41 residue and activation of PAR1 resulting in ubiquitin-driven noncanonical stimulation of p38α MAPK signaling mediated by TAB1 and TAB2 on endosomes (4, 5) (Fig. 1*A*). To identify p38 MAPK targets that mediate thrombin-induced proinflammatory responses, human cultured endothelial EA.hy296 cells were pretreated with SB203580, a p38α and p38β selective inhibitor, or dimethylsulfoxide (DMSO) vehicle control and then stimulated with thrombin to determine changes in protein phosphorylation linked to p38 signaling. Cell lysates were collected from the nonagonist treated, 0 min pretreated DMSO and SB203580 controls, performed with two biological replicates, and three thrombin-stimulated replicates for each condition and phosphopeptides were enriched by TiO2, processed for TMT 10-plex labeling and analyzed by LC-MS2/MS3 to quantify the proteome and phosphoproteome (Fig. 1*B*) (12, 13). We quantified 5491 phosphopeptides representing 2317 phosphoproteins with a false discovery rate of <1% (Dataset S1) (https://massive.ucsd.edu/ProteoSAFe/dataset.jsp?task=e93b9204652341f09400906f12ac3ba8; http://proteomecentral.proteomexchange.org/cgi/GetDataset?ID=PXD018406). The phosphosite distribution was 84.6% phosphoserine, 13.5% phosphothreonine, and 1.9% phosphotyrosine (Fig. 1*C*). The majority of the peptides were phosphorylated at a single site (78.5%) with 19.9% peptides phosphorylated at two sites and considerably fewer (1.6%) peptides phosphorylated at three sites (Fig. 1*D*). Cell lysates from endothelial cells treated with or without SB203580 stimulated with thrombin showed agonist-induced p38 phosphorylation in DMSO control cells that was markedly reduced in cells pretreated with the p38 inhibitor SB203580 (Fig. 1*E*), confirming thrombin-stimulated p38 signaling is inhibited by SB203580 pretreatment in EA.hy926 as we previously reported (4, 6).

### Quantitative phosphoproteomic temporal profiling of thrombin-p38 MAPK signaling and biological functions

**Figure 2:**
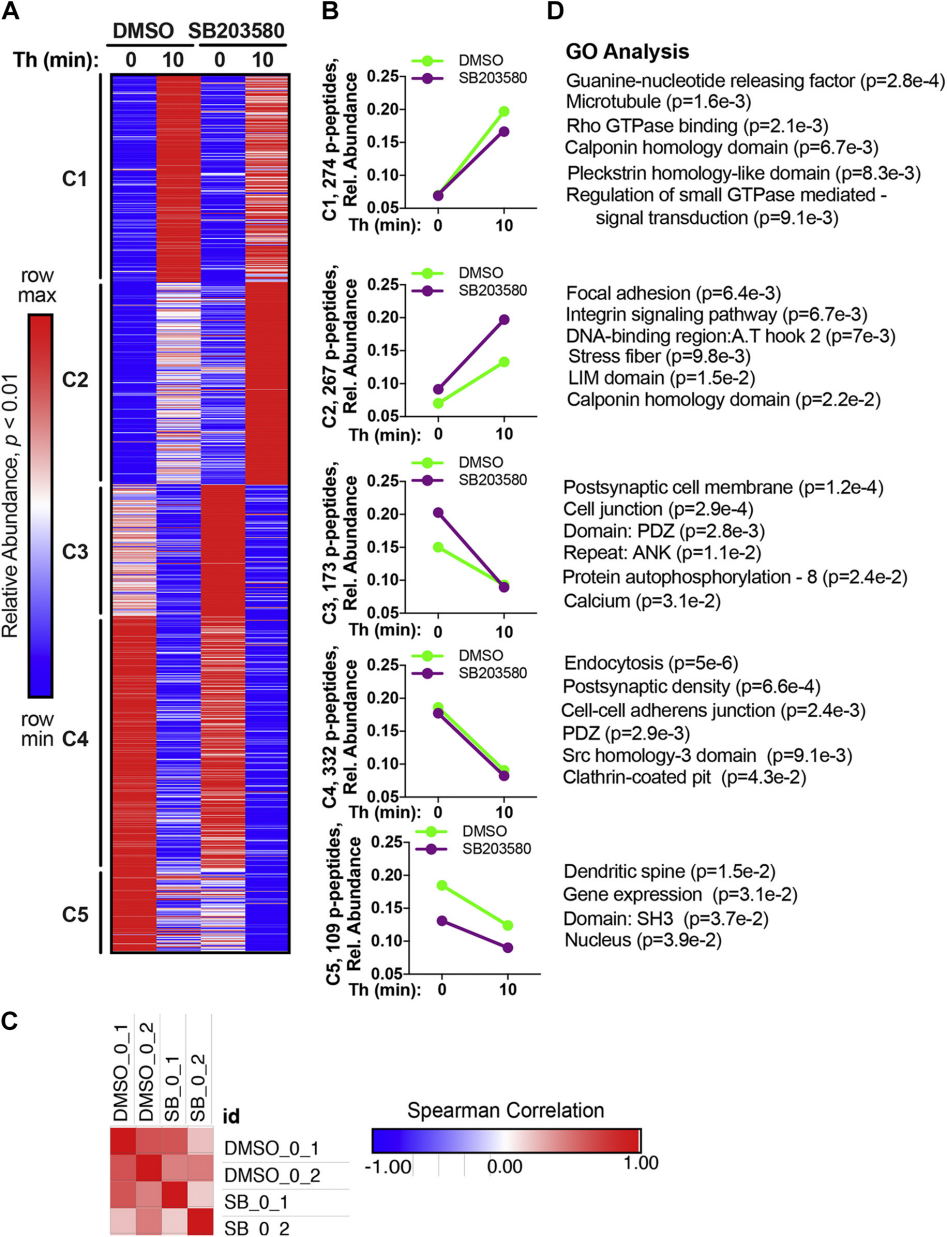
**Quantitative phosphoproteomic temporal profiling of thrombin-p38 MAPK signaling and biological functions.***A*, k-means clustered heat map of the 1155 quantified significantly altered phosphopeptides (p-peptides) (*p* < 0.01) from thrombin (Th) stimulated endothelial cells treated with SB203580 or DMSO separated into five clusters. Increases and decreases in phosphorylation are represented by the *red* and *blue colors*, respectively. Color intensities depict phosphopeptide levels in each sample induced by thrombin normalized to their respective 0 min control and relative (rel.) to the maximum and minimum abundances per row. *B*, Th-induced changes in phosphopeptide abundance in SB203580- and DMSO-treated cells plotted against time. *C*, heat map of Spearman’s correlation coefficients for DMSO and SB203580 0 min replicates analyzed by Morpheus. *D*, gene ontology (GO) enrichment analysis and rank based on statistical significance; *p* values determined by Student’s *t* test. MAPK, mitogen-activated protein kinase.

Temporal changes in the thrombin phosphoproteome in the presence or absence of the SB203580 was evaluated using κ-means clustering of significantly altered phosphopeptides (Fig. 2, *A* and *B*) (Dataset S1 and Dataset S2). Spearman’s correlation coefficients of each of the two 0 min control replicates from DMSO control and SB203580 treatments were compared prior to agonist stimulation and showed positive correlation (Fig. 2*C*), indicating minimal variance of the 0 min control replicates. The thrombin-induced phosphoproteome with and without the p38 inhibitor SB203580 pretreatment separated into five temporally distinct clusters (C) of phosphopeptides. C1 peptides displayed a significant increase in phosphorylation after thrombin stimulation that was reduced by SB203580, whereas C2 peptides showed a more modest significant increase in phosphorylation following thrombin incubation that was markedly enhanced in cells pretreated with the SB203580 p38 inhibitor (Fig. 2, *A* and *B*). Phosphorylation of C3, C4, and C5 peptides was significantly decreased after thrombin stimulation (Fig. 2, *A* and *B*). Inhibition of p38 by SB203580 increased C3 peptide basal phosphorylation at 0 min compared to vehicle control, whereas C5 peptides showed a decrease in basal phosphorylation at 0 min in SB203580 treated cells (Fig. 2, *A* and *B*). In either case, thrombin reduced C3 and C4 peptide phosphorylation, which appeared greater in C3 compared to C5 (Fig. 2, *A* and *B*). No change in phosphorylation was detected in C4 peptides in SB203580-treated cells following thrombin stimulation compared to DMSO control (Fig. 2, *A* and *B*). These data indicate that thrombin stimulates four distinct temporal dynamic changes in protein phosphorylation that are differentially modified by the inhibition of p38 MAPK signaling.

To examine the biological functions associated with the thrombin phosphoproteome regulated by p38 signaling, gene ontology enrichment analysis was used to compare biological processes, cellular compartments, and molecular functions overrepresented in each cluster (Dataset S2) (14, 15). Modulators of small GTPases like Rho were significantly enriched in the C1 cluster including microtubules (Fig. 2*D*). Focal adhesion, integrin signaling, and stress fiber were enriched in C2, whereas the actin-binding calponin homology domain was represented in both C1 and C2 clusters (Fig. 2*D*). Thus, C1 and C2 were enriched in important mediators of endothelial inflammatory responses. C3 was also significantly enriched in the mediators of endothelial proinflammatory responses such as cell junction, PDZ, and ANK domain-containing proteins, autophosphorylation and calcium (Fig. 2*D*). Interestingly, thrombin-modulated C4 phosphopeptides appeared to be independent of p38 activity and were significantly associated with endocytosis, clathrin-coated pit, and modulators of endothelial barrier disruption such as cell–cell adherens junction, PDZ, and Src homology-3 domain (Fig. 2*D*). The C5 cluster having the lowest phosphopeptide representation was enriched in gene expression and nucleus (Fig. 2*D*) and may be attributed to thrombin-stimulated p38-mediated regulation of gene expression (16). Overall, a considerable number of cellular compartments, biological, and molecular functions were associated with endothelial cell activation and barrier disruption (17, 18), consistent with the role for thrombin and p38 in endothelial inflammatory signaling (4, 6).

### Prediction of kinase-specific phosphorylation sites in the thrombin phosphoproteome regulated by p38 MAPK signaling

**Figure 3:**
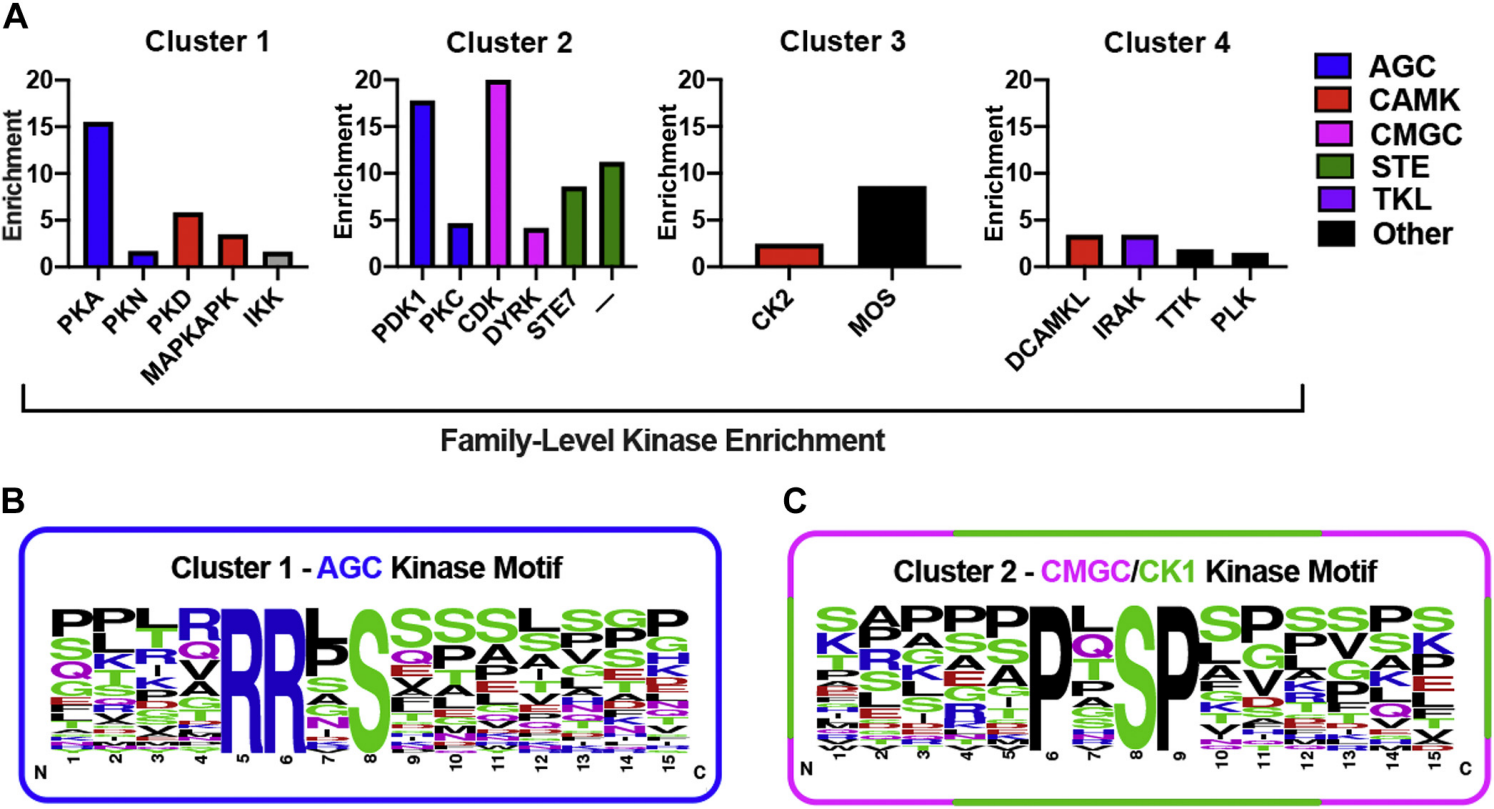
**Predication of kinase-specific phosphorylation sites in the thrombin-induced phosphoproteome regulated by p38 MAPK.***A*, the highly enriched protein kinases predicted for each cluster identified relative to all kinases predicted for the significant phosphopeptides was determined using the GPS tool. The RRxS motif was strongly enriched in Cluster 1 (*B*), and PxSP motif was high abundant in Cluster 2 (*C*) based analysis using motif-X algorithm. The motif logos show the amino acid distributions around the RRxS site of the significant phosphopeptides. GPS, group-based prediction system; MAPK, mitogen-activated protein kinase.

To identify the kinases known or predicted to target proteins for phosphorylation in the specific clusters of the thrombin phosphoproteome regulated by p38, we used group-based prediction system (GPS) algorithm (19) to predict kinase-enriched phosphosites in the five clusters and applied the motif-X algorithm (20) to define the most overrepresented kinase consensus motifs in each cluster (Dataset S3). Using the GPS tool, kinases enriched in each cluster were identified relative to all kinases predicted for the significant phosphopeptides. Enrichment was determined by combining statistical significance with the fold change for the relative proportion of each kinase per cluster. PKA was the most enriched kinase identified in Cluster 1 (Fig. 3*A*). Several other kinases including protein kinase D (PKD) and MAPKAPK (also known as MK2) were also enriched in C1 (Fig. 3*A*). Cluster 2 showed high enrichment of pyruvate dehydrogenase kinase 1 and cyclin-dependent kinase (CDK) (Fig. 3*A*). Kinase enrichment was less evident in C3 and C4 within the experimental time-frame examined (Fig. 3*A*). Using the motif-X algorithm, we found the strongest enrichment was for the RRxS motif in Cluster 1 and for the PxSP motif in Cluster 2 (Fig. 3, *B* and *C*). The motif logos show the amino acid distributions around the RRxS and PxSP consensus sites of the significant phosphopeptides (Fig. 3, *B* and *C*). Other ACG kinases and CMGC kinases were also predicted in Cluster 1 and 2 based on GPS analysis (Fig. 3*A*). The RRxS motif is a PKA consensus phosphorylation site and the PxSP motif is a consensus phosphorylation site for CDKs. Thus, two independent prediction analysis are largely in agreement and consistent with the literature implicating a role for many of these kinases particularly for PKA and CDK in thrombin signaling.

### Thrombin-induced significant and divergent changes in subsets of phosphopeptides following inhibition of p38 activity

**Figure 4:**
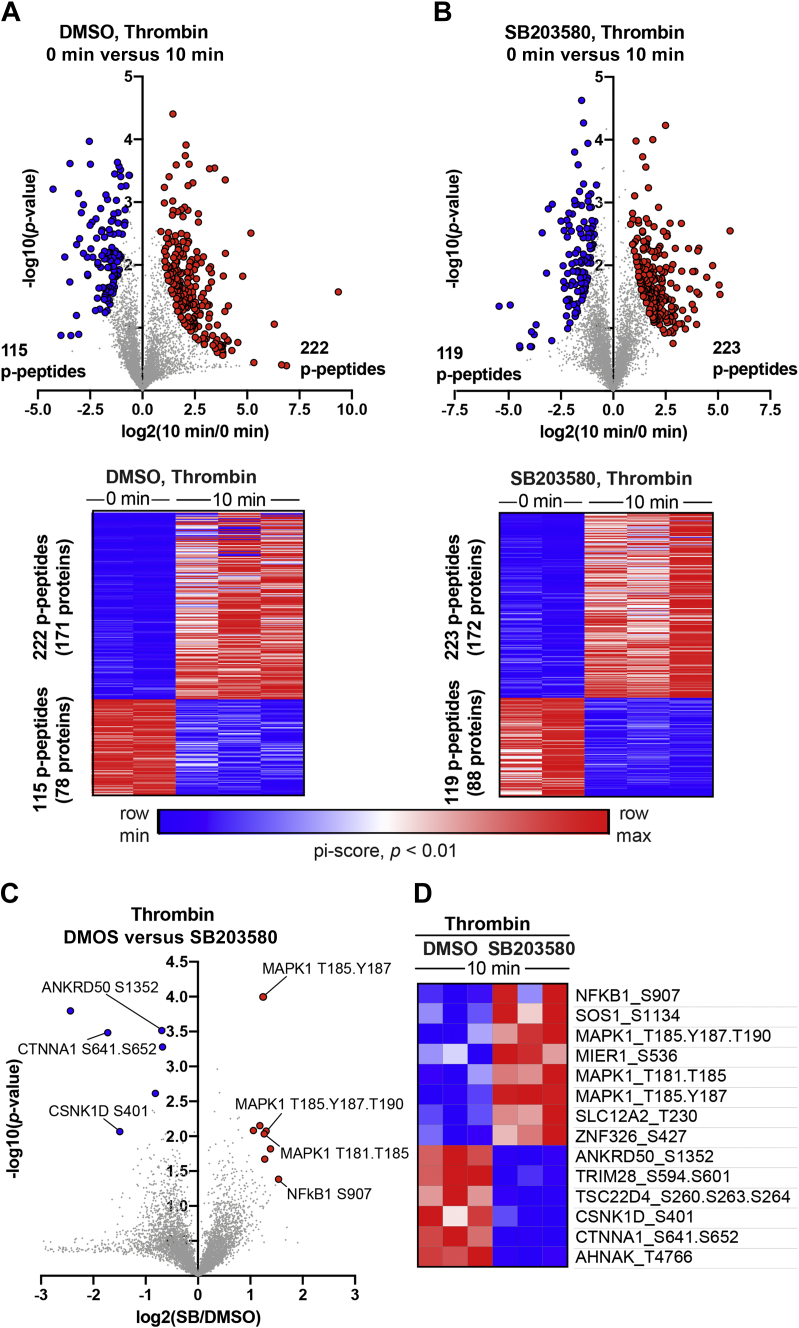
**Thrombin-induced changes in the subsets of phosphopeptides regulated by p38 MAPK signaling.***A* and *B*, volcano plots and heat maps of significantly altered (pi-score, *p* < 0.01) phosphopeptides (p-peptides) from endothelial cells pretreated with DMSO or SB203580 followed by thrombin stimulation. Log-transformed *p* values (Student’s *t* test) associated with individual phosphopeptides are plotted against the log-transformed fold change in abundance between 0 min and 10 min of thrombin stimulation. *C*, volcano plots and heat maps of significantly altered (pi-score, *p* <0.01) phosphopeptides from thrombin-stimulated cells DMSO- *versus* SB203580-treated cells. Log-transformed *p* values (Student’s *t* test) associated with individual phosphopeptides are plotted against the log-transformed fold change in abundance between 10 min of thrombin stimulation in DMSO- *versus* SB203580-treated cells. Color intensities depict changes in phosphopeptide levels relative to the maximum (*red*) and minimum (*blue*) abundances per row. MAPK, mitogen-activated protein kinase.

The effect of thrombin on specific subsets of phosphopeptides was examined using pi-score analysis, which combines statistical significance with agonist-induced fold change in phosphorylation into a single, comparable value (21). In control DMSO-pretreated cells, significant changes in a total of 337 peptides was detected after thrombin stimulation including 222 phosphopeptides from 171 unique proteins that showed increases in phosphorylation and a considerable 115 phosphopeptides from 78 unique proteins displayed decreases in phosphorylation (Dataset S4) (Fig. 4*A*). Cells pretreated with SB203580 and stimulated with thrombin resulted in significant changes in 342 total phosphopeptides, of which 223 phosphopeptides from 172 unique proteins displayed increases in phosphorylation, and 119 phosphopeptides from 88 unique proteins showed decreases in phosphorylation (Fig. 4*B*). The corresponding heat maps of the individual TMT-MS replicates are highly similar (Fig. 4, *A* and *B*), verifying reproducibility of the data.

Next, we examined the effect of p38 inhibition by SB203580 on thrombin-induced changes in a subset of phosphopeptides using pi-score analysis. Eight phosphopeptides with the most significant increases in phosphorylation encode six unique proteins. The six proteins include nuclear factor kappa B subunit 1 (NF-κB1), a key inflammatory mediator; son of sevenless homolog 1 (SOS1), a guanine nucleotide exchange factor for Ras; MAPK1 (also known as ERK2), the key effector of the MAPK/ERK cascade; the transporter solute carrier family 12 member 2 (SLC12A2), and zinc finger protein 326 (ZNF326) that facilitates transcript elongation and alternative splicing (Fig. 4, *C* and *D*). The six phosphopeptides with the greatest significant decreases in phosphorylation represent six unique proteins including ankyrin repeat domain 50 (ANKRD50) a regulator of membrane trafficking and recycling, casein kinase 1 delta (CSNK1D), α-catenin-1 or α-E-catenin (CTNNA1), an adapter that binds to cadherins and facilitates interaction with the actin cytoskeleton, the scaffold neuroblast differentiation-associated protein AHNAK and the transcriptional regulators tripartite motif containing 28 (TRIM28) and transforming growth factor-β-stimulated clone 22 domain family member 4 (TSC22D4) (Fig. 4, *C* and *D*). Heat maps of the corresponding individual TMT-MS replicates are similar indicating high reproducibility of the data (Fig. 4*D*). Together, these findings reveal the identity of several key proteins displaying divergent changes in phosphorylation in response to thrombin and p38 activity. These candidate proteins have not been previously linked to thrombin-induced p38-regulated signaling.

### Thrombin-p38–regulated phosphopeptide site abundance and mapping of key sites in proteins associated with endothelial dysfunction

**Figure 5:**
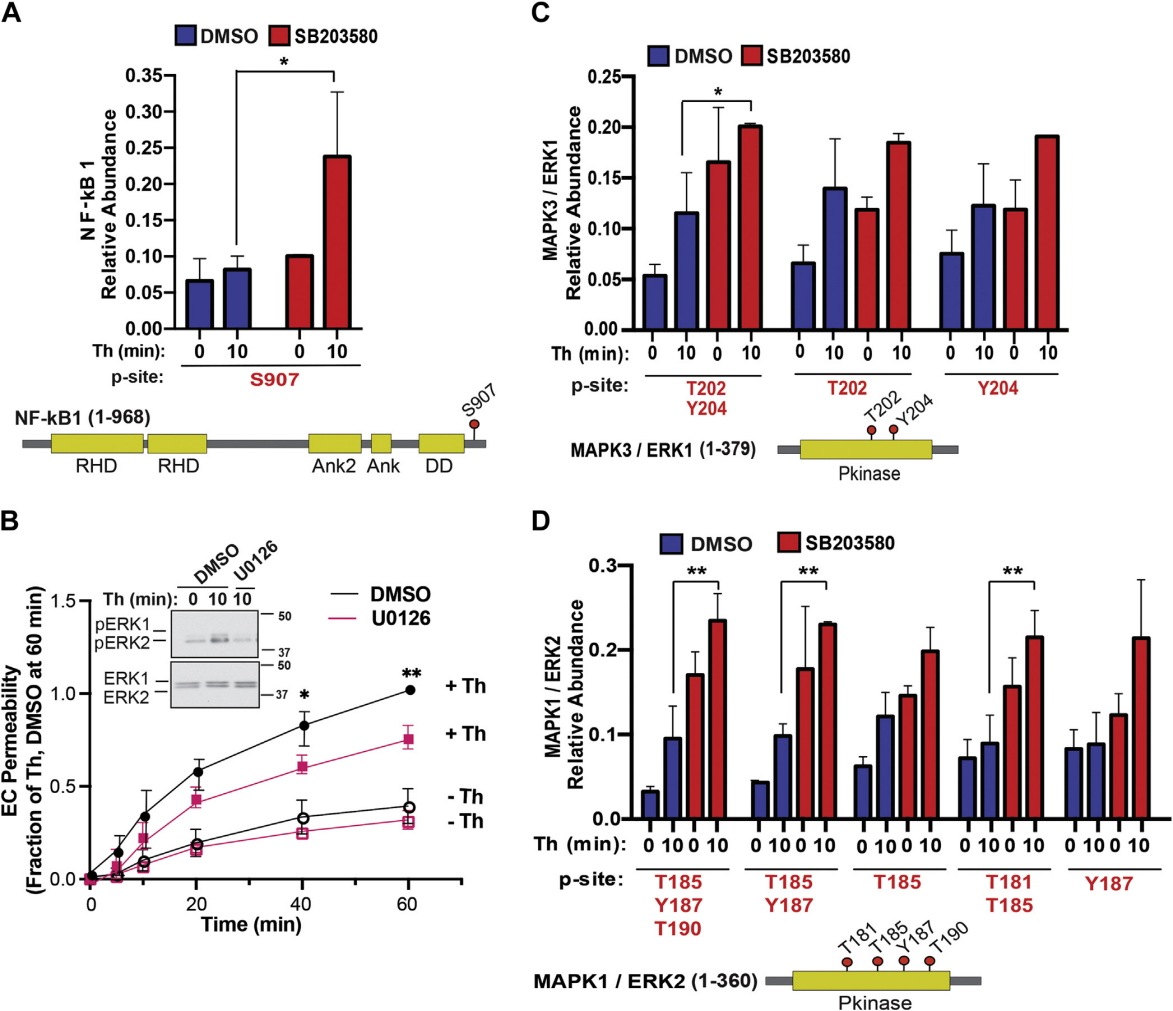
**Thrombin-induced ERK1/2 function in endothelial barrier permeability and thrombin and p38 regulated increases in phosphosite abundance and mapping of key sites in proteins.***A*, *C*, and *D*, bar graphs of quantified significantly altered NF-κB1, MAPK3 (ERK1), and MAPK1 (ERK2) phosphopeptides from thrombin-stimulated cells pretreated with DMSO or SB203580 expressed as relative abundance. Statistical significance was determined by ANOVA (∗*p* < 0.05; ∗∗*p* < 0.01). *Cartoons diagrams* show the protein domain structure and phosphorylation site location, *red dots* with the residue number indicate phosphorylation site. *B*, endothelial cells (EC) pretreated with U0126 or DMSO control were stimulated with thrombin (Th) and barrier permeability monitored over time. The data (mean ± S. D., n = 4) expressed as the fraction of thrombin-induced permeability at 60 min were analyzed by two-way ANOVA (∗*p* <0.05; ∗∗*p* < 0.01). Inset, thrombin-induced phosphorylation (p) of ERK1/2 inhibition with U0126. ERK, extracellular signal–regulated protein kinase; NF-κB1, nuclear factor kappa B subunit 1.

We evaluated thrombin-induced p38-regulated changes in specific phosphosites of key proteins predicted or shown to associate with endothelial dysfunction. The NF-κB transcription factor, formed by the NF-κB1-105 kDa protein in complex with Rel-like domain-containing proteins, is activated by thrombin in endothelial cells and promotes inflammatory responses (22). Phosphorylation of NF-κB1 at the S907 site by thrombin was significantly enhanced in endothelial cells pretreated with the p38 inhibitor SB203580 compared to DMSO control cells with no significant change in basal phosphorylation (Fig. 5*A*). These findings suggest that p38 MAPK may function in a negative feedback loop to attenuate NF-κB1 S907 phosphorylation following thrombin stimulation.

Next, we first established ERK1/2 function in endothelial dysfunction using the U0126 MEK inhibitor, which effectively blocked thrombin-stimulated ERK1/2 phosphorylation in human cultured endothelial cells (Fig. 5*B*, *Inset*). In DMSO control cells, thrombin caused a marked increase in endothelial barrier permeability that was significantly reduced in cells pretreated with U0126, at 40 and 60 min (Fig. 5*B*). These findings indicate that ERK1/2 functions as an effector of thrombin signaling to regulate endothelial barrier disruption. Interestingly, analysis of the thrombin phosphoproteome indicates that inhibition of p38 with SB203580 alone enhanced basal phosphorylation of ERK1 and ERK2 peptides at multiple sites compared to DMSO control cells (Fig. 5, *C* and *D*). Despite increased basal ERK1/2 phosphorylation in SB203580-treated cells, thrombin modestly increased ERK1/2 phosphorylation, at multiple sites including ERK1 T202, Y204, ERK2 T185, Y187, T190, ERK2 T185, Y187, and ERK2 T181, T185 peptides (Fig. 5, *C* and *D*). These findings suggest that p38 may function in multiple negative feedback loops to control changes in NF-κB and ERK1/2 protein phosphorylation, key proteins associated with endothelial dysfunction.

**Figure 6:**
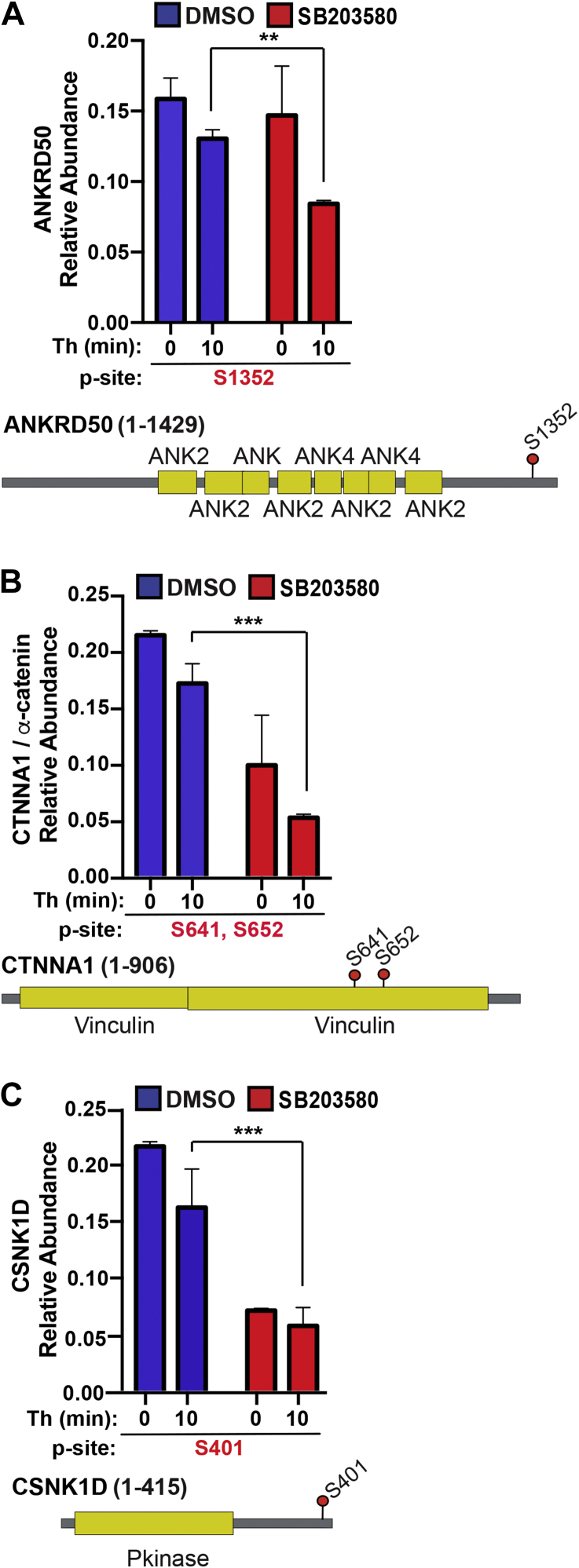
**Thrombin-p38 regulated decreases in phosphosite abundance and mapping of key sites in proteins associated with endothelial dysfunction.***A*–*C*, bar graphs of quantified ANKRD50, CTNNA1 (α-catenin), and CSNK1D phosphopeptides from thrombin-stimulated cells pretreated with DMSO or SB203580 expressed as relative abundance. Statistical significance was determined by two-way ANOVA (∗∗*p* < 0.01; ∗∗∗*p* <0.001). *Cartoons diagrams* show the protein domain structure and phosphorylation site location, *red dots* with the residue number indicate phosphorylation site.

In addition to regulating increases in protein phosphorylation, we examined whether p38 regulated decreases in phosphorylation at specific sites of other key proteins. ANKRD50, a large protein involved in membrane trafficking and recycling (23), is expressed in endothelial cells (24) and exhibited basal phosphorylation at the S1352 site that was not significantly altered in SB203580-treated cells (Fig. 6*A*). However, S1352 phosphorylation was significantly decreased by thrombin in SB203580-treated cells compared to control cells (Fig. 6*A*). The α-catenin protein, a key component of adherens junction that links cadherins to the actin cytoskeleton, showed a marked decrease in phosphorylation of S641 and S652 sites in SB203580-treated cells compared to DMSO control cells (Fig. 6*B*). However, thrombin caused a further modest decrease in α-catenin phosphorylation in control and SB203580-treated cells (Fig. 6*B*). While a role for casein kinase-2 in cadherin phosphorylation has been reported (25, 26), much less is known about the function of casein kinase 1 in endothelial cells except that it can phosphorylate cadherins *in vitro* (27). Our results indicate that inhibition of p38 caused a decrease CSNK1 phosphorylation in both untreated and thrombin-treated cells, suggesting a potential important role for p38 in CSNK1 phosphorylation at the S401 site (Fig. 6*C*). Taken together, these results indicate that p38 activity functions to control both increases and decreases in basal phosphorylation of several key proteins implicated in endothelial dysfunction and in some cases, the p38-dependent changes in protein phosphorylation were further altered by thrombin stimulation.

### p38 negatively regulates ERK1/2 phosphorylation and α-catenin phosphorylation

**Figure 7:**
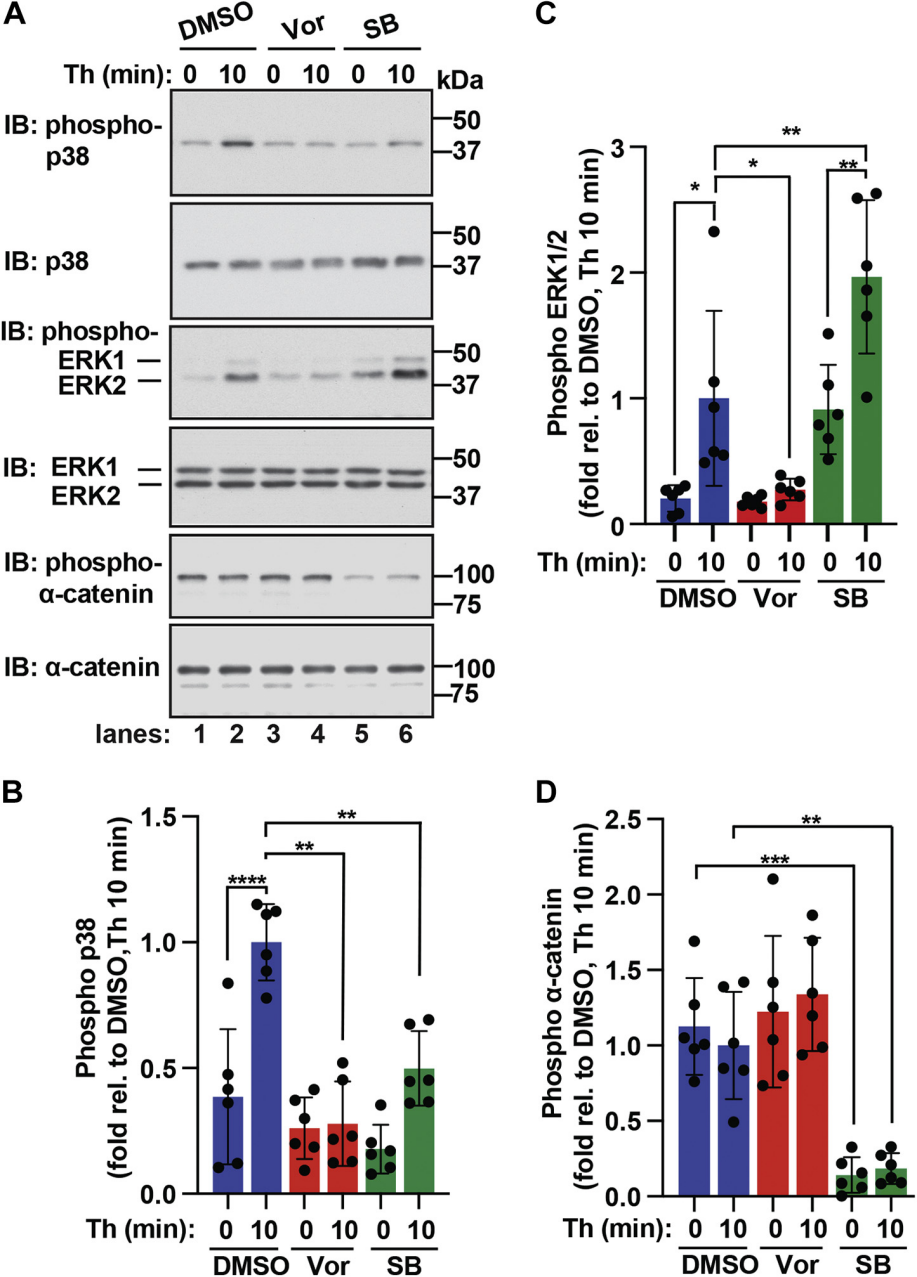
**Thrombin and p38 regulated the modulation of ERK1/2 and α-catenin phosphorylation.***A*–*D*, endothelial EA.hy926 cells pretreated with PAR1 antagonist vorapaxar (vor) or p38 inhibitor SB203580 (SB) were stimulated with thrombin (Th) for 10 min. Equivalent amounts of cell lysates were immunoblotted (IB) as indicated. The data (mean ± S. D., n = 6) are expressed as the fold relative (rel.) to thrombin-stimulated DMSO control cells and analyzed by ANOVA (∗*p* <0.05; ∗∗*p* < 0.01; ∗∗∗*p* < 0.001; ∗∗∗∗*p* < 0.0001). ERK, extracellular signal–regulated protein kinase; PAR1, protease-activated receptor-1.

To validate thrombin and p38 regulation of ERK1/2 and α-catenin phosphorylation, we used a PAR1-selective antagonist vorapaxar and commercially available antibodies that specifically detect phosphorylated ERK1, ERK2, or α-catenin at sites identified in our phosphoproteomic analyses. In these studies, endothelial cells were pretreated with or without the PAR1 antagonist vorapaxar or the p38 inhibitor SB203580 and then stimulated with thrombin. Cell lysates were immunoblotted using a phospho-ERK1/2 antibody that detects ERK1 phosphorylation at T202 and Y204 sites and ERK2 phosphorylation at T185 and Y187 sites and a phospho-α-catenin antibody that detects the S652 phosphorylated site. In control cells, thrombin induced a significant increase in p38 phosphorylation that was significantly reduced in endothelial cells pretreated with vorapaxar (Fig. 7*A*, *lanes 1*–*4*, *top panels*, and *B*), indicating that PAR1 is required for thrombin-stimulated p38 phosphorylation. Similarly, thrombin induced a significant increase in ERK2 phosphorylation with a modest increase in ERK1 phosphorylation detected in control cells that was virtually ablated in cells pretreated with vorapaxar (Fig. 7A, *lanes 1*–*4*, *middle panels*, and *C*), consistent with a role for PAR1. In contrast to ERK1/2, a high basal level of α-catenin S652 phosphorylation was detected even in the absence of thrombin stimulation and was not altered by vorapaxar (Fig. 7*A*, *lanes 1–4, lower panels,* and *D*), suggesting that basal S652 phosphorylation of α-catenin occurs independent of thrombin and activation of PAR1.

Next, we evaluated the effect of p38 inhibition on thrombin-stimulated phosphorylation of ERK1/2 and α-catenin. As expected, inhibition of p38 catalytic activity with SB203580 significantly decreased thrombin-induced p38 phosphorylation compared to control cells (Fig. 7*A*, *lanes 1–2 versus lanes 5–6, top panels,* and *B*), confirming that thrombin/PAR1-stimulated p38 phosphorylation is attributed primarily to autophosphorylation rather than upstream MAPKs in endothelial cells (4, 5). Interestingly, inhibition of p38 with SB203580 resulted in a marked increase in basal ERK2 phosphorylation and a modest increase in basal ERK1 phosphorylation (Fig. 7*A*, *lanes 1–2 versus lanes 5–6, middle panels,* and *C*), consistent with our phosphoproteomic analysis (Fig. 5, *C* and *D*). Regardless, thrombin significantly amplified ERK1 and ERK2 phosphorylation over basal in SB203580-treated cells (Fig. 7*A*, *lanes 2 and 6, middle panels* and *C*). SB203580 inhibition of p38 activity also caused a significant decrease in basal α-catenin S652 phosphorylation in nonagonist-treated cells compared to control cells and was not altered by thrombin stimulation (Fig. 7*A*, *lanes 1–2 versus lanes 5–6, lower panels,* and *D*), similar to that observed in the phosphoproteomic analysis (Fig. 6*B*). These findings indicate that thrombin-induced PAR1-stimulated p38 signaling negatively regulates ERK1/2 phosphorylation, while α-catenin phosphorylation at the S652 site is regulated by p38 activity but not by thrombin-stimulated PAR1 signaling.

### The p38*α* isoform is required for negative regulation of ERK1/2 phosphorylation but dispensable for *α*-catenin basal phosphorylation

**Figure 8:**
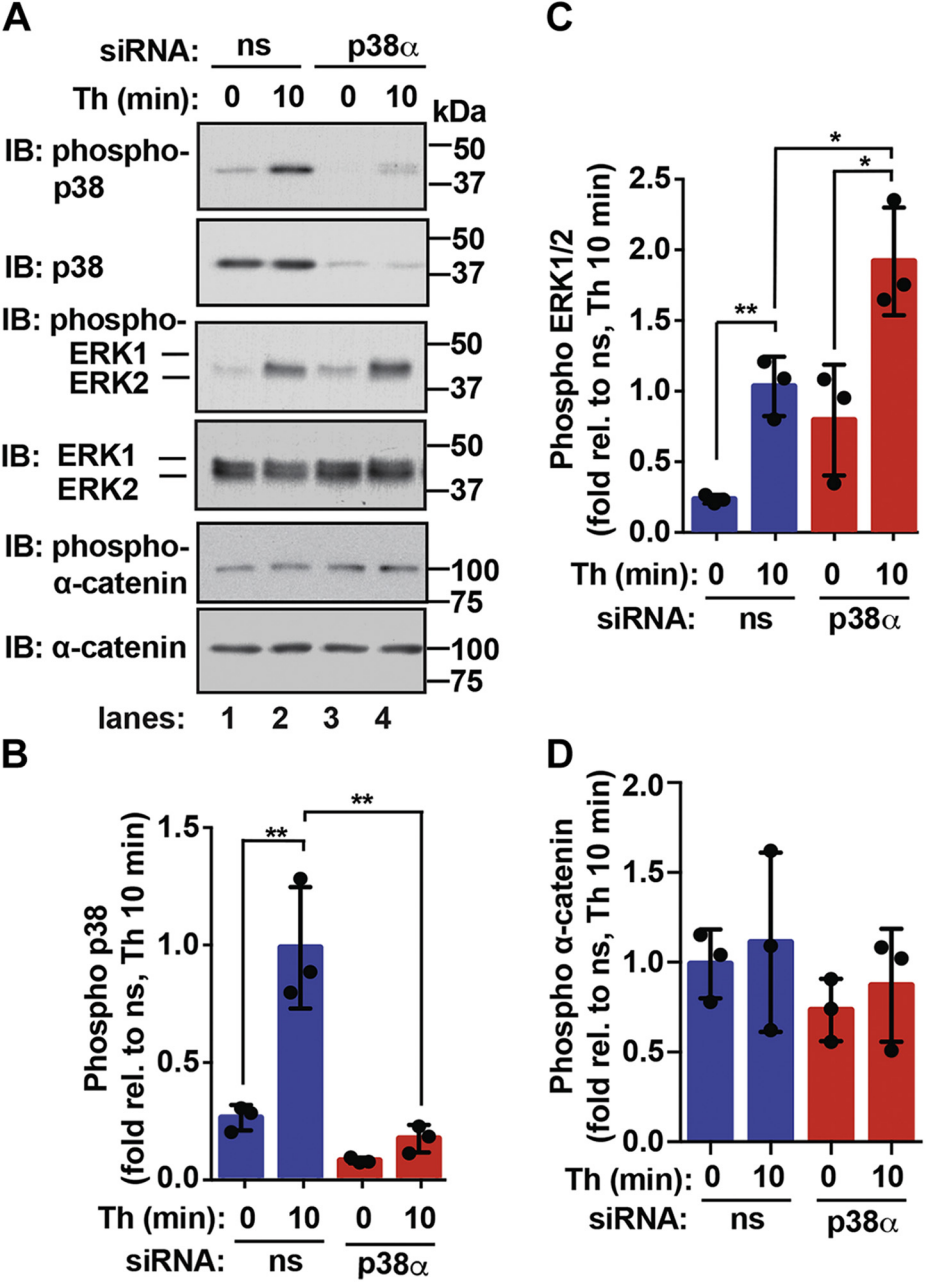
**ERK1/2 and α-catenin phosphorylation by the p38α isoform.***A*–*D*, endothelial EA.hy926 cells were transfected with nonspecific (ns) or p38α specific siRNA and then either left untreated (0 min) or treated with thrombin (Th) for 10 min. Equivalent amounts of cell lysates were immunoblotted (IB) as indicated. The data (mean ± S. D., n = 3) are expressed as the fold relative (rel.) to thrombin-stimulated DMSO control cells and analyzed by ANOVA (∗*p* <0.05; ∗∗*p* < 0.01). ERK, extracellular signal–regulated protein kinase.

**Figure 9:**
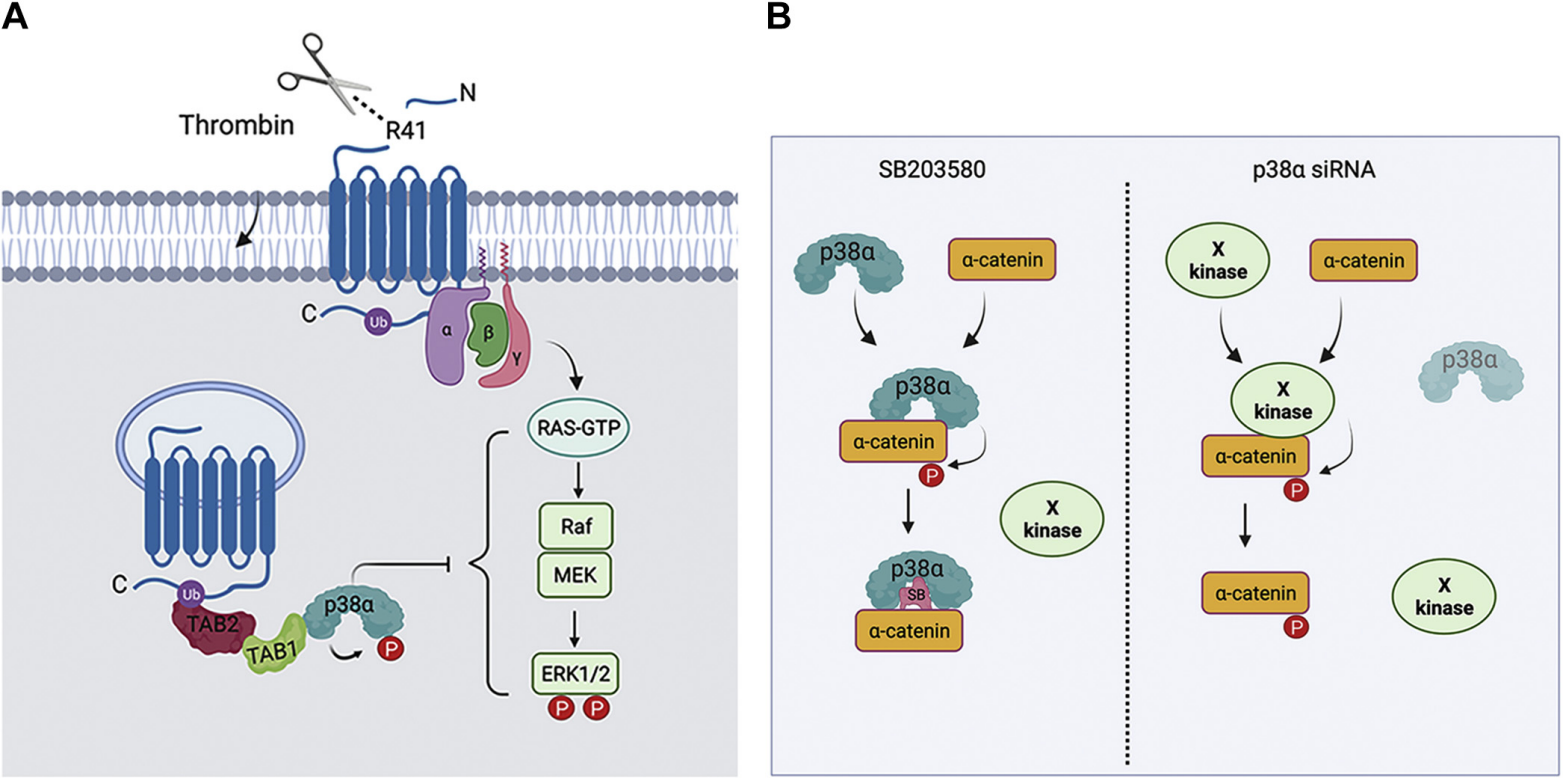
**Model of thrombin and p38 regulation of ERK1/2 and α-catenin phosphorylation**. *A*, thrombin induces p38 activation *via* a noncanonical ubiquitin-driven TAB1/TAB2-dependent pathway, whereas thrombin-activated PAR1 stimulates ERK1/2 activation through a canonical three-tiered kinase cased mediated by G protein-stimulated Ras-Raf-MEK induced ERK1/2 activation. The p38 MAPK negatively regulates ERK1/2 signaling, resulting in amplified ERK1/2 phosphorylation induced by thrombin. However, the mechanism p38 and ERK1/2 of convergence is not known. *B*, inhibition of p38 by SB203580 blocks α-catenin basal phosphorylation at S652. However, depletion of p38α by siRNA fails to affect basal phosphorylation of α-catenin, suggesting that another kinase may function redundantly to phosphorylation α-catenin in the absence of p38α expression. ERK, extracellular signal–regulated protein kinase; PAR1, protease-activated receptor-1; TAB1, transforming growth factor-β-activated kinase 1-binding protein 1.

To examine the function of the p38α isoform in thrombin-stimulated ERK1/2 phosphorylation, siRNA was used to specifically knockdown p38α isoform protein expression in endothelial cells. In nonspecific siRNA-transfected control cells, thrombin induced a significant increase in p38 phosphorylation that was virtually abolished in p38α siRNA–transfected cells depleted of p38 protein expression (Fig. 8*A*, *lanes 1*–*4*, *top panels*, and *B*), suggesting that p38α function as the major effector for thrombin signaling in endothelial cells. In p38α siRNA–depleted cells, basal ERK1/2 phosphorylation was elevated (Fig. 8*A*, *lanes 1–4, middle panels* and B), confirming that p38α functions to negatively regulate ERK1/2 phosphorylation as demonstrated in our phosphoproteomic analysis (Fig. 5, *C* and *D*). However, thrombin significantly amplified ERK1/2 phosphorylation in p38α-depleted cells compared to siRNA-transfected control cells (Fig. 8*A*, *lanes 1–4, middle panels,* and *C*). These results indicate that the p38α isoform contributes to a negative feedback mechanism resulting in amplified ERK1/2 phosphorylation induced by thrombin (Fig. 9*A*). In contrast, depletion of the p38α isoform failed to perturb α-catenin phosphorylation either basally or in thrombin-treated cells compared to nonspecific siRNA-transfected control cells (Fig. 8*A*, *lanes 1–4, lower panels,* and *D*), suggesting that p38α expression is dispensable for basal α-catenin phosphorylation at the S652 site. These data suggest a complex regulation of α-catenin phosphorylation that requires p38α catalytic activity for basal phosphorylation but is not entirely dependent on p38α expression (Fig. 9*B*), suggesting that another kinase may function redundantly in the absence of p38α to phosphorylate α-catenin at the S652 site.

## Discussion

The endothelial barrier is dynamically regulated by multiple intersecting pathways induced by GPCRs. In addition to the well-established thrombin-induced RhoA/MLC pathway, we defined a role for p38 MAPK signaling in thrombin-stimulated endothelial dysfunction that is independent of the RhoA/MLC pathway (4, 6). However, the downstream effectors by which thrombin-stimulated p38 signaling promotes endothelial barrier disruption and other inflammatory responses remains poorly understood. To identify p38-phosphorylated targets induced by thrombin, we performed a comprehensive quantitative phosphoproteomic analysis using human cultured endothelial cells. We found that p38 regulates at least four unique dynamic profiles of the thrombin phosphoproteome. Our study further identified several thrombin- and p38-regulated key proteins associated with endothelial dysfunction and important biological processes including microtubules, focal adhesions, stress fiber, Rho, small GTPases, cell–cell adherens junction proteins, and a subset of kinases. We also discovered that p38 signaling negatively regulates ERK1/2, a mediator of endothelial barrier disruption, resulting in amplified ERK1/2 phosphorylation in response to thrombin stimulation of PAR1. The results also indicate that p38 signaling regulates phosphorylation of α-catenin, a key component of adherens junctions. However, our analysis of the α-catenin S652 site indicated that p38-dependent phosphorylation is independent of thrombin-activation of PAR1. This study reveals a rich array of proteins and pathways associated with thrombin-induced p38 inflammatory signaling in endothelial cells that may serve as potential targets for therapeutic intervention.

Protein phosphorylation is a reversible process and a key mediator of GPCR signal transduction in mammalian cells. Phosphorylation is abundant and regulates the function of most if not all proteins. Despite the extensive study of protein phosphorylation, identified kinases exist for only 5% of the phosphoproteome and the vast majority of phosphosites has not been studied and has no identified function (28). To gain insight into the thrombin-p38 signaling network, we conducted a phosphoproteomic analysis and identified four temporally distinct subsets of phosphopeptides that displayed diverse changes in phosphorylation regulated by p38 and one subset that was not regulated by p38. The temporal and spatial regulation of kinases and phosphatases, the abundance of specific kinases and phosphatases in endothelial cells, as well as the catalytic efficiency of a particular phosphorylation site likely contributes to the diversity of the thrombin phosphoproteome. Of the distinct subsets of phosphopeptides identified, PKA and the PKA consensus site RRXS motif was enriched in Cluster 1. These findings are consistent with previous studies suggesting a role for PKA in negative feedback regulation of thrombin signaling in endothelial cells (29, 30). PKD and MAPKAPK (also known as MK2) were also identified in Cluster 1. PKD has been implicated in thrombin-induced barrier disruption (31) and we recently reported that MK2 functions downstream of thrombin-stimulated p38 signaling to regulate endothelial barrier dynamics (6). Whereas Cluster 2 showed high enrichment of pyruvate dehydrogenase kinase 1 and CDK, both can function downstream of thrombin signaling in different cell types (32, 33). The PxSP motif is a consensus phosphorylation site for CDKs, which was the most enriched in Cluster 2 and is consistent with a role for CDK phosphorylation in thrombin signaling. The global analysis of the thrombin phosphoproteome regulated by p38 provides important information regarding the kinases and target proteins that may mediate endothelial dysfunction.

Cell–cell contacts are formed by adherens junctions and are important for maintaining endothelial barrier integrity. Adherens junctions are dynamic structures composed of transmembrane cadherin proteins that are linked to the actin cytoskeleton through interactions with α- and β-catenins. Interestingly, α-catenin has been shown to function in actin dynamics (34, 35), suggesting that phosphorylation of α-catenin may be functionally important. Indeed, phosphorylation at S641 by casein kinase-2 modulates interactions between α-catenin and β-catenin (36). However, α-catenin phosphorylation at an additional S652 site has been identified by MS studies in various cell types (37, 38, 39), although the functional significance of S652 phosphorylation is not known. In our study, we used MS and showed that p38 signaling positively regulates phosphorylation of α-catenin at two key sites, S641 and S652 (Fig. 6*B*). In validation studies using a commercially available antibody that detects the α-catenin S652 phosphorylation, we found that inhibition of p38 activity with SB203580 virtually ablated basal α-catenin phosphorylation at the S652 site. Unexpectedly, however, we found that depletion of p38α failed to block basal S652 phosphorylation of α-catenin detected in endothelial cells, suggesting that p38α and α-catenin may physically associate and upon depletion of p38α, the S652 site is exposed and phosphorylated by a different kinase (Fig. 9*B*). Since, SB203580 targets both p38α and p38β, it is also possible that p38β is functionally redundant and phosphorylates α-catenin in the absence of p38α, but this remains to be determined.

Unlike α-catenin, we found that inhibition of p38 signaling negatively regulates phosphorylation of ERK1/2, since inhibition of p38 with SB203580 resulted in a significant increase in basal phosphorylation at multiple ERK1/2 phosphosites detected by MS (Fig. 5, *C* and *D*) and in cell lysates using immunoblot analysis of ERK1/2 phosphorylation (Figs. 7 and 8). Despite the increase in basal phosphorylation upon p38 inhibition, thrombin significantly amplified ERK1/2 phosphorylation (Figs. 7 and 8). While we show that ERK1/2 mediates thrombin-induced endothelial barrier disruption (Fig. 5*B*), consistent with its role in endothelial inflammation (40, 41), it remains to be determined how crosstalk between p38 and ERK1/2 signaling impacts endothelial dysfunction in response to GPCR stimulation. The precise regulation of ERK1/2 signaling is critical for controlling important cellular functions. Thus, multiple feedback mechanisms have evolved to control the spatial and temporal aspects of ERK1/2 signaling and are mediated primarily by phosphorylation of various pathway components (42). ERK1/2 activation by GPCRs occurs through a three-tiered kinase cascade initiated by heterotrimeric G proteins and numerous effectors that stimulate activation of the small GTPase Ras, which initiates the phospho-relay pathway (Fig. 9*A*) (43). Activated Ras feeds into c-Raf signaling, the upstream MAP3K, that phosphorylates MEK1,2, the MAP2K, which mediates phosphorylation of ERK1 at T185 and Y187 and ERK2 at T202 and Y204 sites. Interestingly, previous work demonstrated that activated p38α binds directly to ERK1/2 and correlates with the inhibition of ERK1/2 activity (44), suggesting that p38α binding may sterically block MEK1 phosphorylation. However, the mechanism by which thrombin-stimulated p38α activity regulates ERK1/2 phosphorylation *via* the Ras-initiated three-tiered kinase cascade is not known.

In summary, the current study provides a global analysis of the thrombin phosphoproteome regulated by the p38 MAPK signaling and critical information regarding the proteins and pathways that mediate endothelial dysfunction. Clearly, future studies are needed to interrogate the function of p38 and ERK1/2 crosstalk in GPCR-induced endothelial dysfunction and the role of the p38-regulated α-catenin S652 phosphorylation in endothelial barrier disruption. In addition, the mechanism by which p38 negatively regulates thrombin-induced ERK1/2 phosphorylation is not known and important to determine. Finally, this study has identified numerous other proteins likely to have important functions in GPCR- and p38-induced endothelial dysfunction that may serve as targets for therapeutic intervention.

## Experimental procedures

### Reagent and antibodies

Human α-Thrombin was from Enzyme Research Laboratories (#HT1002a). The following antibodies were from Cell Signaling Technologies including p44/42 MAPK ERK1/2 (#9102), phospho-p44/42 MAPK ERK1/2 (#9106), p38 MAPK (#8690), phospho-p38 MAPK (#4511), α-E-catenin (#3236), and phospho-α-E (Ser652)-catenin antibody (#13061). Horseradish peroxidase-conjugated goat-anti rabbit (#1706515) and goat-anti mouse antibodies (#1706516) were from BioRad. Vorapaxar or SCH 530348 (#1755) was from Axon MedChem, SB203580 (#S8307) was from Sigma-Aldrich, and U0126 (#U-6770) was from LC Laboratories.

### Endothelial cell culture and treatments

Human umbilical vein endothelial cell–derived EA.hy926 cells were (45) were obtained from American Type Culture Collection, grown and cultured per the manufacturer’s instructions, expanded and frozen at passage 2. Endothelial EA.hy926 cells were used for MS–based proteomics and all other experiments from passage 2, up to passage 6. Endothelial cells were pretreated with 10 μM vorapaxar for 1 h or 5 μM SB203580 for 30 min at 37 °C. Cells were then stimulated with 10 nM thrombin for 10 min at 37 °C or left untreated 0 min and processed as described below.

### Endothelial barrier permeability

Endothelial EA.hy926 cells were seeded into 12-well plates with transwell inserts at 1.75 × 10^5^ cells per well and grown for 72 h and then pretreated with or without 100 nM U0126 or DMSO vehicle control for 30 min at 37 °C prior to 10 nM thrombin treatment. Diffusion of Evans Blue bound to bovine serum albumin was used to monitor barrier disruption and quantified over a 1 h time-course by measuring the A_605_ using a Molecular Devices SpectraMax ABS Plus 384 Microplate Readers as previously described (4, 12).

### Cell transfections with siRNA

Endothelial EA.hy926 cells were seeded on 24-well plates at 1.7 ×10^5^ cells per well, grown overnight, and then transfected with siRNAs using Trans-IT X2 (Mirus, MIR 6000) per the manufacturer’s instructions. The siRNAs used in these studies. AllStars negative control nonspecific siRNA (#1027281) and p38α-specific siRNA 5′-AACTGCGGTTACTTAAACATA-3′ were purchased from Qiagen.

### Immunoblotting

Endothelial EA.hy926 cells were seeded into 24-well plates at 1.7 ×10^5^ cells per well and transfected. Serum-starved cells were then either left untreated or treated as described above, lysed, and protein concentrations determined using a bicinchoninic acid protein assay (ThermoFisher Scientific). Equivalent amounts of cell lysates were resolved by SDS-PAGE, transferred to PVDF membranes, and incubated with antibodies as indicated. Membranes were developed by chemiluminescence and quantified by densitometry using NIH ImageJ software or analyzed using a ChemiDoc Imaging System (BioRad).

### Mass spectrometry

#### Cell treatments and lysis

EA.hy926 cells were grown in 150 cm^2^ dishes, serum starved overnight, pretreated with DMS0 vehicle control or 5 μM SB203580 for 30 min, and then stimulated with or without 10 nM Thrombin for 10 min at 37 °C. Cells were washed with 1X PBS and lysed in a buffer containing 75 mM NaCl, 3% SDS, 1 mM NaF, 1 mM β-glycerophosphate, 1 mM NaVO_4_, 1 mM NaPP, 1 mM PMSF, 1X complete EDTA-free protease inhibitor cocktail and 50 mM Hepes (13). Cells were sonicated to ensure full lysis, and cellular debris was removed *via* centrifugation (16,000*g*, 10 min, 4 ^°^C)

#### Protein digestion

Proteins were denatured by addition of urea (4 M final concentration), then reduced and alkylated with DTT and iodoacetamide, respectively (46). The denatured, alkylated proteins were then methanol/chloroform precipitated. Proteins were resolubilized in 1 M urea in 50 mM Hepes, pH 8.5 and digested in a two-step process (LysC – 16 h; Trypsin – 6 h) before being desalted with C18 Sep-Paks (47). Digested peptides were quantified using a bicinchoninic acid assay. Peptides from matched samples were aliquoted for both standard proteomics (50 μg) and phospho-proteomics (2 mg).

#### Phosphopeptide enrichment

Phosphopeptides were enriched by TiO2 beads as previously described (48). Briefly, the following buffers were made. Binding buffer: 2 M lactic acid, 50% acetonitrile; wash buffer: 50% acetonitrile/0.1% TFA; and elution buffer: 50 mM KH_2_PO_4_, pH 10. TiO2 beads were washed (once with binding buffer, once with elution buffer, and twice with binding buffer). Peptides were resuspended in binding buffer, mixed with beads at a ratio of 1:4 (peptides to beads), and vortexed at room temperature for 1 h. Beads were then washed three times with binding buffer, followed by three times with wash buffer. Phosphopeptides were eluted from the beads with elution buffer (two 5 min incubation while vortexing). Enriched peptides were desalted with C18 Sep-Paks, then lyophilized and stored at −80 °C until they were labeled for quantitation.

#### TMT labeling

Samples were labeled with TMT 10-plex reagents (49, 50) for multiplexed quantitative proteomics. TMT reagent 126 was reserved for a “bridge channel”, and the remaining reagents were used to label digests in random order. Bridge channels consisted of an equal amount of each sample pooled together. The bridge served as a means to control for experimental variation between MS experiments. Labeling was conducted for 1 h at room temperature and quenched by the addition of 9 μl of 5% hydroxylamine. Samples were then acidified by the addition of 50 μl of 1% TFA and pooled. The pooled, multiplex samples were desalted with C18 Sep-Paks.

#### Basic pH reverse-phase liquid chromatography fractionation

Fractionation was carried out by basic pH reverse-phase liquid chromatography(47). Briefly, samples were solubilized in 110 μl of 5% formic acid in 5% acetonitrile, and 100 μl was separated on a 4.6 mm × 250 mm C18 column on an Ultimate 3000 HPLC. The resultant 96 fractions were combined into 24 distinct fractions and dried prior to multiplexed LC-MS3 analysis.

#### LC-MS3 analysis–MS data acquisition

Labeled peptides were resuspended in 5% acetonitrile/5% formic acid and analyzed on an Orbitrap Fusion Tribrid mass spectrometer with an in-line Easy-nLC 1000 System. All data acquired were centrioded. Samples were loaded onto a 30 cm in-house pulled and packed glass capillary column (I.D. 100 μm, O.D. 350 μm). The column was packed with 0.5 cm of 5 μm C4 resin followed by 0.5 cm of 3 μm C18 resin, with the remainder of the column packed with 1.8 μm of C18 resin. Following sample loading, peptides were eluted using a gradient ranging from 11 to 30% acetonitrile in 0.125% formic acid over 85 min at a flow rate of 300 nl/minute and heating the column to 60 °C. Electrospray ionization was assisted by the application of 2000 V of electricity through a T-junction connecting the column to the nLC.

MS1 spectra were acquired in data-dependent mode with a scan range of 500 to 1200 m/z and a resolution of 60,000. Automatic gain control (AGC) was set to 2 × 10^5^ with a maximum ion inject time of 100 ms and a lower threshold for ion intensity of 5 × 10^4^. Ions selected for MS2 analysis were isolated in the quadrupole at 0.5 Th. Ions were fragmented using collision-induced dissociation, with a normalized collision energy of 30% and were detected in the linear ion trap with a rapid scan rate. AGC was set to 1 × 10^4^ and a maximum inject time of 35 ms. MS3 analysis was conducted using the synchronous precursor selection option to maximize TMT quantitation sensitivity (51). Up to 10 (regular proteomics) or 3 (phospho-proteomics) MS2 ions were simultaneously isolated and fragmented with high energy collision–induced dissociation using a normalized energy of 50%. MS3 fragment ions were analyzed in the Oribtrap at a resolution of 6 × 10^4^. The AGC was set to 5 × 10^4^ using a maximum ion injection time of 150 ms. MS2 ions 40 m/z below and 15 m/z above the MS1 precursor ion were excluded from MS3 selection.

#### Peptide identification by proteome discoverer

Resultant data files were processed using Proteome Discoverer 2.1. MS2 data were queried against the Uniprot human database (downloaded: 05/2017; 59,010 entries) using the Sequest algorithm (52). A decoy search was also conducted with sequences in reversed order (53, 54, 55). For MS1 spectra, a mass tolerance of 50 ppm was used and for MS2 spectra, a 0.6 Da tolerance was used. Static modifications included TMT 10-plex reagents on lysine and peptide n-termini and carbamidomethylation of cysteines. Variable oxidation of methionine and for the phospho-proteomics experiments, phosphorylation of serine, threonine, and tyrosine residues were also included in the search parameters. The enzyme specificity was set to full trypsin digest with two missed cleavages permitted. The data were filtered to a 1% peptide and protein-level false discovery rate using the target-decoy strategy (53, 54, 55).

#### Normalization and data analysis

Reporter ion intensities were extracted from MS3 spectra for quantitative analysis. For the regular proteomics, protein-level quantitation values were calculated by summing signal to noise values for all peptides per protein meeting the specified filters. The data were normalized in a two-step process as previously described (56). First, the values for each protein were normalized to the pooled bridge channel value of all the samples in the experiment. Then, the values were normalized to the median of each reporter ion channel. Phosphopeptide normalization was performed similarly, except quantitation was summed to the unique phosphopeptide level, then normalized to the total protein level. Phosphosite localization was performed using the PhosphoRS node within Proteome Discoverer. Phosphosites with a PhosphoRS score of >50% were considered confidently localized. The *PTMphinder* R package was used to localize phosphorylated residues in the context of their corresponding full-length proteins and extract flanking sequences prior to motif analysis (57). Prior to direct statistical comparisons, K-means clustering was used to group all quantified phosphopeptides with similar temporal profiles. The optimal number of clusters was determined using the elbow method. Gene ontology analysis was used to identify enriched pathways in clustered phosphopeptides (14, 15). Kinase prediction was performed using GPS 5.0 (19) and motif consensus site enrichment analysis was performed using the *motif-x* R package (20). To identify significant phosphopeptide changes, treatment conditions were compared using a Student’s *t* test with Welch’s correction if the variances were unequal. The pi-score method was used to prioritize significantly altered phosphopeptides (21). Spearman’s correlation coefficients were determined by Morpheus. The color intensity depicts the Spearman’s correlation coefficient range of −1.0 to 1.0. Values between 0 and 1.0 (red intensity) indicate positive association, 0 (white) indicates no association, and 0 to −1.0 (blue intensity) indicates negative association.

### Model and prediction analysis

Schematics were created in Adobe Illustrator and Photoshop. The cartoons in Figures 1 and 9 were created with BioRender.com.

### Statistical analysis

Data were analyzed using Prism 7.0 statistical software, Microsoft Excel, R and Morpheus (https://software.broadinstitute.org/morpheus). Statistical analysis methods are indicated in the Figure legends.

## Data availability

The mass spectrometry data have been deposited on MassIVE (MSV000085235) and on the ProteomeXchange (PDX018406).

## Supporting information

This article contains supporting information.

## Conflict of interest

The authors declare that they have no conflicts of interest with the contents of this article.
